# Influence of socio-economic status on habitual physical activity and sedentary behavior in 8- to 11-year old children

**DOI:** 10.1186/1471-2458-10-214

**Published:** 2010-04-27

**Authors:** Clemens Drenowatz, Joey C Eisenmann, Karin A Pfeiffer, Greg Welk, Kate Heelan, Douglas Gentile, David Walsh

**Affiliations:** 1 Department of Kinesiology, Michigan State University, East Lansing, MI, USA; 2 Department of Pediatrics and Human Development, Michigan State University, East Lansing, MI, USA; 3 Department of Kinesiology, Iowa State University, Ames, IA, USA; 4 Department of Health and Physical Education/Human Performance Lab, University of Nebraska-Kearney, Kearney, NE, USA; 5 Department of Psychology, Iowa State University, Ames, IA, USA; 6 National Institute on Media and the Family, Minneapolis, MN, USA

## Abstract

**Background:**

While socio-economic status has been shown to be an important determinant of health and physical activity in adults, results for children and adolescents are less consistent. The purpose of this study, therefore, is to examine whether physical activity and sedentary behavior differs in children by socio-economic status (SES) independent of body mass index.

**Methods:**

Data were from two cohorts including 271 children (117 males; 154 females) in study 1 and 131 children in study 2 (63 males; 68 females). The average age was 9.6 and 8.8 years respectively. Height and body mass were assessed according to standard procedures and body mass index (BMI, kg/m^2^) was calculated. Parent-reported household income was used to determine SES. Habitual, free-living physical activity (PA) was assessed by a pedometer (steps/day) in study 1 and accelerometer (time spent in moderate-to-vigorous PA) in study 2. Self-reported time spent watching TV and on the computer was used as measure of sedentary behavior. Differences in PA and sedentary behavior by SES were initially tested using ANOVA. Further analyses used ANCOVA controlling for BMI, as well as leg length in the pedometer cohort.

**Results:**

In study 1, mean daily steps differed significantly among SES groups with lower SES groups approximating 10,500 steps/day compared to about 12,000 steps/day in the higher SES groups. These differences remained significant (p < 0.05) when controlling for leg length. Lower SES children, however, had higher body mass and BMI compared to higher SES groups (p < 0.05) and PA no longer remained significant when further controlling for BMI. In study 2 results depended on the methodology used to determine time spent in moderate-to-vigorous physical activity (MVPA). Only one equation resulted in significant group differences (p = 0.015), and these differences remained after controlling for BMI. Significant differences between SES groups were shown for sedentary behavior in both cohorts (P < 0.05) with higher SES groups spending less time watching TV than low SES groups.

**Conclusions:**

Children from a low SES show a trend of lower PA levels and spend more time in sedentary behavior than high SES children; however, differences in PA were influenced by BMI. The higher BMI in these children might be another factor contributing to increased health risks among low SES children compared to children from with a higher SES.

## Background

Socio-economic status (SES) is an important determinant of health and well-being because it influences people's attitudes, experiences, and exposure to several health risk factors [1]. Indeed, several studies have shown that low socioeconomic characteristics (e.g., household income, education level) are related to a variety of chronic diseases and all-cause mortality (e.g., health disparity) [2,3]. The relation between low SES and health is not limited to adults, as children who grow up in a low SES family have a higher risk for an unhealthier lifestyle and cardiovascular disease (CVD) compared to children from higher SES [1,3].

Habitual physical activity is considered to be essential for optimal physical growth and development of the child [4,5]. Despite the widely known benefits of physical activity, many young people do not meet recommended levels of physical activity [6]. Furthermore, several studies show an inverse relationship between physical activity levels in youth and parental educational level [1,7-9]. Others have also found that parental income is inversely related to sedentary behavior such as time spent watching television (TV) [10-12]. Despite these findings, Raudsepp [13] points out that the relationship between SES and physical activity has not been well quantified since physical activity levels are typically assessed by self-report/recall. Recent studies using objective measurements (e.g., accelerometry) have reported equivocal findings [14-16]. Another important methodological consideration when examining the relation between SES and physical activity behavior is that obesity is more prevalent in low SES children [17,18]. However, most of the previous studies have not considered this factor, thus confounding the results between SES and physical activity/sedentary behavior.

Gorely et al. [11] also emphasized the importance of sedentary behavior when examining health risk factors in children and adolescents, but acknowledged that the relationship between sedentary behavior and health outcomes is less clear than the relationship between health risks and physical activity. Similarly, Katzmarzyk et al. [19] point out that sedentariness is a unique human behavior that may represent a different paradigm than that associated with lack of physical activity. Their results show that increased sitting time increases the risk of mortality independent of physical activity levels. The higher risk was explained by adverse changes in cardiovascular function and glucose tolerance. In addition it has been suggested that sitting or sedentary behavior alters lipoprotein lipase activity in several tissues and may be related to cellular signals and physiological responses in relation to prolonged sitting and other sedentary behaviors [20]. Finally, a variety of studies showed that sedentary behavior tracks better than physical activity from childhood into adolescence [21,22], which further undermines the importance of examining sedentary behavior separately from physical activity.

To better understand the physical activity phenotype and successfully promote a physically active lifestyle among children and adolescents, more information on the impact of socio-economic factors on physical activity levels and sedentary behavior in children is needed [23]. Further, this study, will consider the relationship between BMI and physical activity and sedentary behavior, which has not been addressed sufficiently in previous studies. In this paper, data from two cohorts is utilized to examine whether objectively measured physical activity and/or sedentary behavior in children differs between socio-economic groups as determined by household income, independent of adiposity.

## Methods

Data from two separate studies were used to examine whether household income influences physical activity levels and sedentary behavior in children. Habitual, free-living physical activity was assessed objectively in both studies (pedometer in study 1 and accelerometer in study 2). Sedentary behaviors (watching TV and using the computer) were assessed by self-report.

### Subjects

#### Study 1

Data for study 1 were obtained as part of a multi-level intervention study (SWITCH) aimed at obesity prevention [24]. The project involved six public elementary schools in a midwestern U.S. community (Cedar Rapids, IA, USA) and data were collected during the Fall of 2006. A total of 897 children were invited to participate in the study of which 583 enrolled (65% response rate). The data herein were collected prior to subject knowledge of randomization into control or treatment groups; thus, the data analysis can be considered as a cross-sectional, observational study design. Parental consent and child assent were obtained prior to data collection. The study protocol was approved by the University of Minnesota and Iowa State University Institutional Review Boards and is in accordance with the Declaration of Helsinki. Due to non-compliance with pedometer wear time or missing physical activity data the sample size used for this analysis included 271 youth (117 males; 154 females) with a mean age of 9.6 ± 0.9 years. There were no significant differences in age, BMI, and income between compliant and non-compliant children. The majority of the subjects were Caucasian (88.0%) with the remainder of the sample consisting of African-Americans (3.3%), Hispanics (1.5%) and other (7.3%). Due to missing data in additional covariates, the sample size varies by analysis (see results).

#### Study 2

Data for Study 2 were obtained from a mixed-longitudinal study of the development of adiposity and blood pressure. The project has followed a small cohort of youth from a rural midwestern U.S. community (Kearney, NE, USA) and the present data were obtained in the summers of 2006 and 2007. The total number of subjects invited to participate in this study is difficult to ascertain since the recruitment was through newspaper advertisements, word of mouth, etc. Hence, it could be estimated from census data that about 2000 3-8 year old children reside in Kearney, NE. In this study, 174 children participated. To more closely resemble the sample from study 1 all subjects under the age of 6 years were excluded from data analysis. This resulted in a total of 131 children (63 males; 68 females) who completed a laboratory visit in one of the two years. All subjects provided compliant accelerometry data. The mean age of the sample was 7.8 ± 2.3 years and the majority were Caucasian (93.7%). The study was approved by the University of Nebraska, Kearney and Michigan State University Institutional Review Boards and is in accordance with the Declaration of Helsinki. Informed consent was obtained from all participating parents and assent was obtained from participating children.

### Household income

Household income was used as the indicator of SES and was obtained by self-report from the parent(s)/guardian(s) on a demographic and health survey. Ferreira et al. [10] reported that family income is a consistent correlate of PA in children and adolescents. In both samples, categories were established using annual household income with the income categories varying by study. In study 1, the sample was divided into five groups by annual household income (< $ 24,999; $ 25,000 - $ 35,999; $36,000 - $ 54,999; $ 55,000 - $ 100,000; > $ 100,000). Due to the smaller sample size, subjects in study 2 were grouped into three groups (low/< $ 50,000; medium/$ 50,000 - $ 75,000; high/> $ 75,000).

### Habitual physical activity

Physical activity was assessed with different measures in the two samples but similar protocols and processing strategies were used to analyze the data. In Study 1, participants wore a pedometer (Digiwalker 200-SW) for a seven day period and recorded steps each day. The Digiwalker is one of the most commonly used pedometers and has been shown to be among the most accurate [25]. The subjects were given instructions on wearing the pedometer, and all pedometers were tested using the established 20-step test prior to data collection [26]. The first day of data collection was not used since it did not include a full day and helped to eliminate the threat of reactivity. Subjects recorded the time on/time off and number of steps accumulated over the subsequent 7-day period. Participants were included in the analysis only if they had at least 4 days (3 weekday and 1 weekend) with the pedometer worn for at least 10 hours per day.

In study 2, participants wore an Actigraph GT1M (Fort Walton, FL) for seven consecutive days. The Actigraph is a widely used accelerometer that has been shown to have utility for evaluating physical activity in youth [27,28]. For the current study, a one-minute epoch was used. Children were required to wear the Actigraph for at least three full weekdays and one weekend day to be included in the analysis. To ensure that the data reflected actual levels of physical activity in the participants, detailed screening procedures were conducted to detect non-compliance with the protocol. Data for each day were flagged if more than 3 instances of 20 periods of consecutive zeros were detected during this time frame. The data for these flagged days were then recoded as 'missing'. To be considered a full day, the monitor had to be worn for at least 480 minutes (8 hours). If the criteria were not achieved, then that particular day was excluded from the data analysis and any participant with more than 3 missing days was removed from analysis. Parents/guardians were asked to fill out a daily log sheet in conjunction with each child wearing the physical activity monitor to determine when the monitor was removed for bathing, swimming or forgotten. To ensure accuracy, each day of the minute-by-minute physical activity data were downloaded and manually checked against the daily physical activity log sheet. Summing the 24, 60-minute time blocks comprising each day, generated daily total counts. Activity counts were converted to counts/min based on the total daily time the unit was worn. Moderate to vigorous physical activity (MVPA) was calculated as the total amount of time each day spent in moderate and vigorous activity using two different calibration equations [27,29], since there is currently no consensus on the 'gold standard' equation [30]. In addition, this approach allows for comparison to other studies which use either equation. In one equation MVPA was determined using an age-specific MET equation for activity counts: MET = 2.757 + (0.0015 × counts/min) - (0.08957 × age (yr)) - (0.000038 × counts/min × age (yr)) [27]. A MET value above 6 was considered to be moderate to vigorous. The second equation used a cutpoint developed through a calibration study that employed direct observation as the criterion measure [29]. Children were observed while going through a series of free-living activities. The cutpoint was determined using receiver operator characteristic (ROC) curves that identified the count that most accurately corresponded with the observed transition to moderate physical activity (the threshold of MVPA). The process yielded a value of 2172 counts/min with reported sensitivity and specificity of 95.9 and 87.6, respectively [29].

### Sedentary behavior

In both studies, time spent on the computer and time watching TV were recorded. Given the age of the subjects, parent report was used for younger children and the child report for older children in study 2 but all children reported screen time in study 1. Total screen time as well as time spent watching TV and at the computer separately was recorded in hours per week. Due to a possible limitation in access to a computer, time spent watching TV was used as measure for sedentary behavior.

### Anthropometry

In both studies, chronological age (yrs) was determined as the decimal age (observation date minus birthdate). Standing height (cm) and body mass (kg) were measured according to standard procedures [31]. In study 1, sitting height was also assessed and subtracted from standing height to estimate leg length which was used a covariate in the analysis of pedometer steps. The body mass index (BMI, kg/m^2^) was calculated from measurements of standing height and body mass.

### Statistical analysis

Descriptive statistics were calculated for the anthropometric, physical activity, and sedentary behavior characteristics in both samples. Differences in physical activity levels and sedentary behavior by household income were initially tested using univariate analysis of variance (ANOVA). Further analyses used ANCOVA controlling separately for sex and BMI, since both sex [31-34] and body fat [35,36] influence physical activity. In study 1, leg length was considered as an additional covariate, since pedometers were used to assess physical activity and individuals with longer legs cover the same distance with fewer steps [37]. In study 2 accelerometers were used so it was not necessary to control for additional covariates. All statistical analyses were conducted using SPSS (Version 16.0). Due to the homogeneity of age across the subgroups in either study, age was not considered as a covariate in the analysis.

## Results

### Study 1

Descriptive characteristics of the subjects participating in study 1 are shown in Table 1. The majority (57%) of the participants lived in a household with an annual income above $55,000 with 20% having an annual income above $100,000. Roughly 25% of the participants lived in a household with an annual income below $36,000 with 13% below $25,000. The remaining 18% had an annual household income between $36,000 and $50,000. In the total sample, there were no significant group differences across income categories for age (total mean 9.6 ± 0.9), height (total mean 138.5 ± 8.1), or leg length (total mean 65.7 ± 5.1). Although not statistically significant, it may be important to note that the small mean differences in height reflect to some degree the small differences in age (e.g., 9.8 yrs v. 9.4 yrs and 140 cm v. 137 cm, respectively). Body mass and BMI were significantly different across income groups (*F*(4,266) = 5.186, p = 0.001) with participants from lower income groups being heavier than those from higher income groups. Differences remained significant after controlling for age and sex (F(4,266) = 4.645, p = 0.001). While overweight rates, based on BMI (85^th ^- 95^th ^percentile), were quite similar across income groups (range 20% - 24%), obesity rates were lower in higher SES groups. The two lowest income groups with 24% and 23% of children classified as obese were significantly different from the highest income group with only 2% of obese children (p = 0.007 and 0.024).

**Table 1:** Descriptive Statistics for Subjects from Study 1. Values are Mean (SD).

	< 24,999 $	25,000 - 35,999 $	36,000 - 54,999 $	55,000 - 100,000 $	> 100,000 $	Total
Number of Subjects (male/female)	36 (9/27)	30 (10/20)	50 (23/27)	100 (48/52)	55 (29/26)	271 (119/152)

Chronolog. Age (yrs.)	9.8 (1.0)	9.7 (0.9)	9.6 (0.9)	9.6 (0.8)	9.4 (1.0)	9.6 (0.9)

Height (cm)	140.1 (7.8)	139.3 (8.4)	139.0 (7.4)	138.0 (8.8)	137.4 (7.3)	138.5 (8.1)

Leg Length (cm)	66.0 (4.4)	66.1 (5.3)	66.0 (4.3)	65.6 (6.0)	65.2 (4.6)	65.7 (5.1)

Body mass (kg) *	41.5 (13.1)	39.4 (10.8)	38.6 (10.5)	35.6 (10.1)	33.6 (7.1)	36.9 (10.5)

BMI (kg/m^2^) *	20.8 (5.3)	20.1 (4.1)	19.8 (4.0)	18.6 (3.7)	17.6 (2.4)	19.1 (4.0)

Overweight (%)	24.3	23.3	20.0	23.0	20.0	22.1

Obese (%)	24.3	23.3	14.0	8.0	1.8	11.8

Physical Activity (Steps/day) *	10935 (3138)	10535 (3226)	10710 (3654)	12038 (3244)	12270 (2665)	11518 (2356)

Time spent watching TV (hrs/week) *	23.7 (18.4)	36.7 (19.2)	22.7 (18.8)	22.5 (16.2)	19.8 (13.6)	23.7 (17.4)

Total Screen time (hrs/week) *	37.9 (29.6)	54.0 (29.3)	38.7 (29.0)	38.0 (30.0)	29.9 (21.2)	38.2 (28.6)

* Significant group difference (ANOVA with p = 0.05)

**Figure 1:**
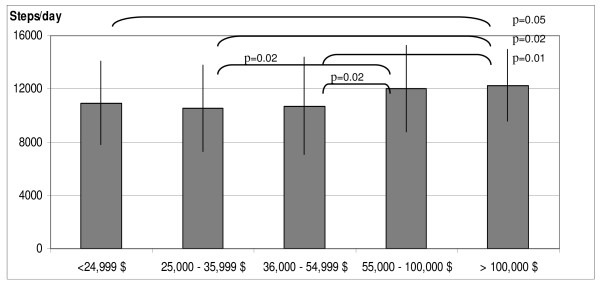
**Mean daily steps by household income (Study 1) -- landscape format**.

**Figure 2:**
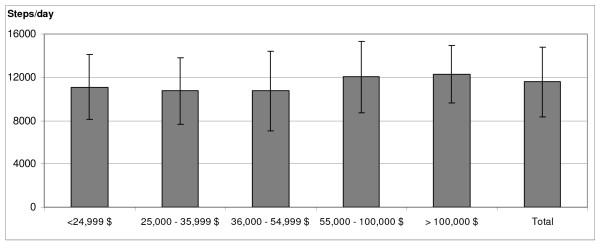
**Mean Daily Steps by Income Groups controlling for BMI (Study 1) -- landscape format**.

Figure 1 shows that the mean steps per day were significantly different across income groups (*F*(4, 271) = 3.28, p = 0.01). Post-hoc analysis revealed significant differences in physical activity between the highest income group and the three lowest income groups. In general, mean steps per day approximated 12,000-12,200 in the higher income groups and 10,500-10,900 in the lower income groups. These differences remained significant when controlling for leg length (*F*(4, 268) = 2.86, p = 0.02), but were not significant when controlling for BMI (*F*(4, 265) = 1.52, p = 0.196)(Figure 2). Sex differences in physical activity were found as well with boys (12,086+3981 steps per day) taking more steps per day than girls ((10,053+2734 steps per day) (p = 0.001)), thus sex was also entered as a covariate. Differences in mean daily steps between income groups were no longer significant when controlling for sex; although there was a trend towards significance (*F*(4,262) = 2.17, p = 0.073). Similarly, group differences were removed when controlling for sex and leg length (*F*(4,260 = 2.11, p = 0.081). Finally, there was no significant difference in daily steps by income group when controlling for BMI and sex (*F*(4, 257) = 2.04, p = 0.09).

**Figure 3:**
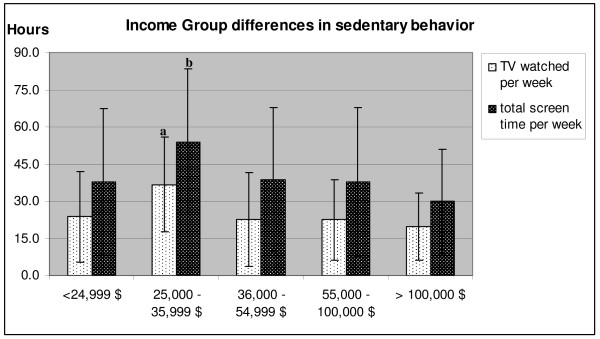
**Differences in weekly hours watching TV and total screen time by household income (Study 1)**. a. time spent watching TV significantly different from all other groups. b. total screen-time significantly different from highest income group only.

The results concerning sedentary behavior revealed significant differences in weekly hours of TV watching (*F*(4, 263) = 5.30, p = 0.001) with the second lowest income group showing higher amounts of watching TV than all other groups (Figure 3). The second lowest income group had also significantly higher values than the highest income group in total screen time (p = 0.003) (Figure 3). Differences remained significant when controlling for sex (p = 0.006), BMI (p = 0.027), and sex and BMI combined (p = 0.012).

### Study 2

Table 2 shows the physical characteristics and physical activity levels of the participants in study 2. There were more girls (68%) than boys (32%) in the lowest income group; while the medium and high income group consisted of about half boys (52% and 55%, respectively) and half girls. Sex differences, however, were not significant between SES groups (F(2, 129) = 1.58, p = 0.209). Based on household income data, 21% of the subjects were classified as low SES, 36% were categorized as medium SES, and 43% were classified as high SES. There were no significant group differences for ethnicity, age (total mean 8.8 ± 1.7), height (total mean 127.2 ± 14.3), leg length (total mean 65.7 ± 5.1), body mass (total mean 27.5 ± 9.3), and BMI (total mean 16.5 ± 2.6) between income groups. A higher prevalence of overweight and obese children occurred in the low SES group (11.1% overweight, 14.8% obese) compared to the medium (9.3% overweight, 4.7% obese) and high SES group (4.8% overweight, 6.5% obese).

**Table 2:** Descriptive Statistics for Subjects from Study 2. Values are Mean (SD).

	< 50,000 $	50,000 -- 75,000 $	> 75,000 $	Total
Number of Subjects (male/female)	34 (11/23)	52 (27/25)	45 (25/20)	131 (63/68)

Chronolog. Age (yrs.)	8.6 (1.6)	8.7 (1.7)	8.9 (1.8)	8.8 (1.7)

Height (cm)	124.9 (14.4)	126.8 (14.8)	128.7 (13.8)	127.0 (14.4)

Body mass (kg)	27.9 (11.9)	27.1 (9.4)	27.6 (7.9)	27.5 (9.5)

BMI (kg/m^2^)	17.2 (3.7)	16.4 (2.5)	16.3 (2.1)	16.6 (2.7)

Overweight (%)	11.1	9.3	4.8	7.5

Obese (%)	14.8	4.7	6.5	7.5

MVPA_FM (min/day)	179.7 (83.7)	162.7 (56.6)	187.9 (74.5)	178.0 (71.8)

MVPA_WEL (min/day) *	54.5 (28.7)	49.7 (22.0)	65.0 (29.5)	57.9 (27.8)

Time spent watching TV (hrs/week) *	17.5 (8.5)	15.4 (8.2)	12.5 (5.5)	14.9 (7.4)

MVPA_FM: time spent at moderate-to-vigorous physical activity based on equation by Freedson et al. [18]

MVPA_WEL: time spent at moderate-to-vigorous physical activity based on equation by Welk [20]

* Significant group difference (ANOVA with p = 0.05)

**Figure 4:**
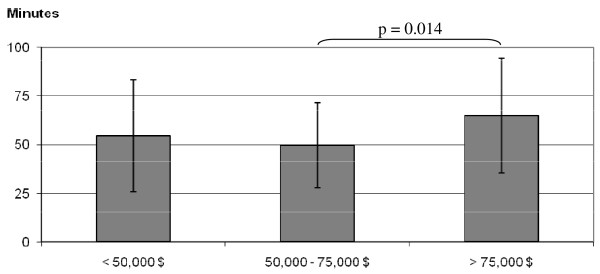
**Differences in minutes spent in accelerometer-determined moderate-to-vigorous physical activity (using the Welk **[27]** equation) by income groups (Study 2)**.

**Figure 5:**
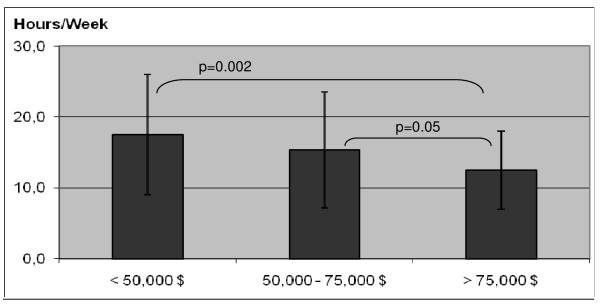
**Differences in weekly hours watching TV by household income (Study 2)**.

Differences in PA only occurred when Welk's [29] equation was used to determine time spent at MVPA (F (2, 129) = 4.32, p = 0.015) (Figure 4). These differences, however, remained when controlling for BMI, sex, and sex and BMI combined (F (2, 129) = 3.91, p = 0.023). There were also significant differences in sedentary behavior by income groups. Although no differences were shown for time spent on the computer, the highest income group displayed significantly lower amounts of hours of watching TV (*F*(2, 129) = 7.02, p = 0.001) (Figure 5). This result remained significant when controlling for sex (p = 0.001), BMI (p = 0.005), and sex and BMI combined (p = 0.004).

## Discussion

Despite the mixed results between the two cohorts, some general conclusions can be drawn. Both studies showed lower physical activity levels in the lower SES groups when BMI was not controlled statistically. However, the differences in physical activity levels were no longer significant after accounting for BMI. Differences in sedentary behavior were evident as well with lower income groups reporting more time spent watching TV, and this difference remained significant after controlling for BMI.

The results are consistent with previous studies that have shown equivocal findings concerning the relationship between SES and physical activity levels in children and adolescents [4,16,22,29-39]. It is likely that the mixed results are due to the difficulties in quantifying both SES and physical activity. However, recent studies [16,37,40], including the present study, using objective assessments of physical activity (e.g., accelerometer, pedometer) still report equivocal results.

There are several possible reasons for differences in habitual physical activity to exist across socio-economic backgrounds, including behavioral, socio-cultural, and/or biological factors. For example, socio-environmental influences may include accessibility to sports/exercise facilities as well as safety [41]. Kantomaa [23] also showed that children in a household with a higher annual income were more involved in club sports, which provides additional opportunities for these youth to be active. However, Macintyre and Mutrie [42] argue that SES does not influence overall physical activity levels in children and adolescence despite a higher participation in formal sports in children and adolescents with a higher SES. They showed that total energy expenditure was not higher in higher SES youth, due to lower participation in unstructured activities. Unfortunately, total energy expenditure is not considered in most studies, including the present paper. The previous studies, however, did not consider BMI in the statistical analysis. Our results clearly indicate the influence of BMI on this relationship.

Besides the often cited socio-environmental reasons, biological aspects have also been shown to influence habitual physical activity [43,44]. Several physiological substances (e.g. estrogen, leptin, ghrelin and orexin) have been shown to influence physical activity, leading to the idea that a "central activity stat" regulates physical activity levels [43,44]. Even though most of these studies have used animal models to determine the role of biological constraints on physical activity, the general assumptions seem to be true for humans as well [37]. Although there is no direct evidence to link these biomarkers with low SES and physical activity, it is possible that they might contribute to higher levels of adiposity. Indeed, BMI is inversely related to SES [45-47]. Our results show that considering BMI is an important covariate in the relationship between SES and physical activity. Specifically, we demonstrated that differences in physical activity levels were no longer evident after controlling for BMI. Even though differences in BMI across SES groups have been reported [47-49], previous studies did not consider this covariate in their analysis.

Generally, a higher BMI is related to lower physical activity levels [50,51]; although cross-sectional studies cannot determine the directionality of this relation it can be argued that decreased physical activity levels are a response to weight gain rather than a contributor [52]. Several studies suggest that maternal under- or overnutrition can induce permanent changes in metabolism and neuroendocrine structure and function that predispose the offspring to energy imbalance and obesity [53-55]. It is possible that events linked to the prenatal environment including pre-pregnancy overweight and excessive weight gain during pregnancy are linked to a disruptive hormonal mileau of the developmental biology, which are associated with appetite, energy balance, and the pathophysiology of obesity [53], which, in turn, could potentially cause lower physical activity levels. In relation to SES, low SES mothers and their offspring are disadvantaged in all of these aspects of health care, environmental exposure, and health behavior [56]. On the other hand, physical activity has been shown to prevent or delay the onset of overweight [57-59], which would suggest that reduction in physical activity precedes excessive weight gain.

The consistent finding across both cohorts in this study was that sedentary behavior (e.g. TV, screen time) was higher among lower SES groups. Previous studies [11,23,42] have also shown an inverse relationship between SES and sedentary behavior but did not control for BMI. Considering BMI as a covariate might influence the results since BMI is related to screen time [60]. It has also been argued that higher BMI levels in low SES adolescents are mediated by screen time [60]. In our study, differences among SES groups in sedentary behavior remained significant after controlling for BMI, which suggests an independent relationship between screen time and SES. It should also be considered that physical inactivity is not necessarily correlated with physical activity even though environmental constraints, like accessibility to facilities and safety to play outside might influence sedentary behavior [11,20,60,61]. Results of the current study also highlight the independency of physical activity and sedentary behavior. Despite no significant differences across SES groups in physical activity when controlling for BMI in study 1, results for sedentary behavior remained significant after controlling for BMI.

Given the differential findings and constructs of physical activity (exercise) and sedentary behavior, it has been posited that inactivity physiology (e.g., the investigation of cellular signals and physiological responses in relation to prolonged sitting and other sedentary behaviors) needs to be considered independent of physical activity [52]. For example, Blanc [62] showed a reduced sensitivity to leptin and insulin with physical inactivity despite no changes in body weight in humans. In addition, lipoprotein-lipase, an important enzyme for lipid metabolism, has been shown to be reduced following prolonged sedentary behavior [62]. This in turn could increase the risk for metabolic diseases like obesity or type 2 diabetes. Even though the research on sedentary behavior is less extensive than that for physical activity, it could be argued that sedentary behavior, just like physical activity, is influenced by environmental and biological constraints. The previously discussed physiologic adaptations in utero and during early childhood could therefore not only influence physical activity, but also sedentary behavior.

Finally, it should also be considered that despite using objective measurements for physical activity, total energy expenditure and nutritional intake were not assessed in either of the two cohorts used in this paper. These, however, are important factors when examining body weight. Interestingly, differences in physical activity levels among SES groups when controlling for BMI remained significant when examining frequency and intensity (accelerometry) while differences in physical activity were no longer significant when only frequency (pedometer) was considered. Another aspect is the different sample size of the study, which did not allow for creating similar SES groups. In addition both studies had a cross-sectional design. Further, it can be argued that SES was determined by household income alone with no information on the size of the household income. Braveman et al. [63] suggest using a variety of components to assess SES including income, wealth, education, occupation, and neighborhood socioeconomic characteristics. When examining the available data on parental education in relation to household income in both cohorts a significant positive relationship between household income and educational level was shown, which supports the use of household income as a single indicator to determine SES. In addition previous studies have shown a strong relationship between physical activity levels or sedentary behavior and household income [10,12].

## Conclusions

In summary, the results show that low SES children are likely to display lower physical activity levels, engage in more sedentary activities and have a higher BMI. Although differences in physical activity were no longer present when controlling for BMI, results for sedentary behavior were not altered after adjusting for BMI. Results of the current study emphasize that physical activity during childhood and adolescence is a complex, multi-factorial phenotype. SES seems to be one aspect related to physical activity and sedentary behavior. BMI, however, is also correlated with SES so untangling these associations is complicated. It is, therefore, important that the interaction between various risk factors related to cardiovascular disease are considered in further examining the problem of health behavior in low SES youth. Longitudinal studies are needed to provide a better understanding of the causal relationship between SES, BMI, sedentary behavior and physical activity during childhood which could contribute to a higher success-rate of interventions. Regardless of the causal pathway, this study shows the importance of targeting low SES youth in intervention programs to enhance health behaviors. Future studies should also consider biological aspects like the pre-natal environment and maternal behavior as well as post-natal influences on physical activity and inactivity. Due to the complex interaction of constraints mentioned in this paper it is important to consider a variety of covariates whenever physical activity and/or sedentary behavior are examined.

## Competing interests

The authors declare that they have no competing interests.

## Authors' contributions

DG and DW coordinated data collection and data management of the SWITCH data set. KH coordinated data collection and data management of the UNK data set. CD and JCE carried out data analysis. KAP and GW helped considerably to draft the manuscript. All authors read and approved the final manuscript.

## Pre-publication history

The pre-publication history for this paper can be accessed here:

http://www.biomedcentral.com/1471-2458/10/214/prepub
